# Death of T. Romeyn Beck, M. D.

**Published:** 1855-12

**Authors:** 


*Death of T. Romeyn Beck, M. D.*—This distinguished man died at Albany, on the 20th of November. At the time of his death he was Secretary to the Board of Regents of the University of the State of New York, and held a professorship in the Albany Medical College. His best fame is perhaps derived from his work on Medical Jurisprudence, which has been honored by several reprints in Europe.

